# Varanid Teeth Asymmetry and Correlation to Body Size

**DOI:** 10.3390/jdb13010009

**Published:** 2025-03-10

**Authors:** Guy Sion, Domenic C. D’Amore

**Affiliations:** 1 Department of Science, The David Yellin Academic College of Education, Jerusalem 9103501, Israel; 2 Gulbali Institute, Charles Sturt University, Boorooma Street, Locked Bag 588, Wagga Wagga, NSW 2678, Australia; 3 Department of Natural Sciences, Daemen University, Amherst, NY 14226, USA; ddamore@daemen.edu

**Keywords:** dentition, directional asymmetry, lizard, Komodo dragon, Varanidae, *Varanus*

## Abstract

Stressors such as injuries, embryonic instability during development, and higher levels of stress hormones such as testosterone can result in increases in fluctuating asymmetry in reptiles and other vertebrates. Digit asymmetry, digit ratio variability, and skull trait asymmetry such as eye and jaw size have been correlated with stress level in both snakes and lizards. Teeth asymmetry has also been used as a biomarker for stress and brain laterality. Body size is correlated with many potential stressors, yet there has been little research on how body size in reptiles relates to asymmetry. We investigate teeth asymmetry within the lizard family Varanidae, a clade with a diverse range of sizes consisting of the largest living lizard, *Varanus komodoensis*. Using a landmark/semi-landmark analysis, we derived Centroid Size for 671 pairs of teeth from 13 varanid species, and asymmetry was derived for each pair. Right-biased asymmetry was significantly greater in the upper tooth row, but breaking up tooth positions into further sections did not yield a significant difference. We found a significant positive linear correlation between body size and right-biased teeth directional asymmetry within *Varanus*, but only when excluding *V. komodoensis*. This significant correlation may result from fewer potential predators and more potential food items, thus resulting in less overall stress. When analyzed separately, *V. komodoensis* individuals with <180 mm head length demonstrated a positive, yet non-significant, trend along a similar trajectory to their congenerics with a high goodness of fit. On the other hand, individuals > 180 mm showed a high degree of scatter, with several specimens having pronounced left-biased asymmetry. We suspect that this dramatic change was due to a combination of ontogenetic niche shift, bigger home ranges, a greater susceptibility to negative anthropogenic influences, and/or a male bias in the bigger specimens sampled, but a larger sample size is required to determine if there is statistical significance in these intra-specific trends. Body asymmetry can reflect brain laterality, which may be a potential driver for the teeth asymmetry seen here.

## 1. Introduction

### 1.1. Stress and Asymmetry

Left-biased asymmetry, where a morphological trait is bigger on the left side than the right, has been correlated with several factors that indirectly reflect higher stress levels. One of the most commonly studied stressors in reptiles are injuries, and specifically injuries to the tail. The effect of stress is reflected in the biomarkers of digit asymmetry [1,2] and a 2:4 digit ratio [3,4]. Skull traits, such as eye size asymmetry, were also correlated with tail injury in both snakes [5] and lizards [6,7], as well as jaw size asymmetry in snakes [5].

Environmental conditions may also be stressors that can manifest in asymmetry. The higher stress can be a result of higher predation risk, and the tail injuries mentioned above can reflect predation risk [7]. Kark [8] compared the mid-digit asymmetry in partridge populations, observing a gradient from core populations in optimal conditions to sub-optimally positioned peripheral populations.

Risk-taking strategy may be derived from brain laterality (defined as brain asymmetry) and mediated by testosterone, which is why higher-risk behaviors and asymmetric digit ratio are correlated with asymmetric behaviors such as footedness [3]. Testosterone is a stress hormone in both humans [9] and lizards [10], and high-risk strategies include behaviors such as spending less time undercover [7]. Sion [7] demonstrated that asymmetric biomarkers may reflect higher testosterone levels, and in particular display left dominance. These conditions were also correlated with high-risk strategies in geckos.

### 1.2. Dental Asymmetry

Dental asymmetry, the deviation from bilateral symmetry in teeth, has been the subject of increasing interest in the fields of dentistry, genetics, and developmental biology [11]. The use of teeth asymmetry to measure environmental instability is not new, but has been explored mainly in humans [12,13,14,15,16,17,18,19]. In humans, teeth asymmetry was more left-biased [20]. Although it is not always significantly correlated with stress, this is argued to be due to a small sample size [21]. While it is often considered a minor aesthetic concern in humans, recent studies suggest that it may be linked to more profound biological processes in vertebrate fetal development such as embryonic exposure [11] and brain laterality [11,12,13,14,15,16,17,18,19]. Teeth asymmetry can indicate asymmetric feeding behavior reflected in the patterns of dental asymmetric wear [22,23], or in tooth emergence and eruption. The latter may provide information on prenatal and early postnatal growth and development [24]. Heikkinen et al. [24] also argued that asymmetrical tooth eruption is not only due to fluctuating asymmetry, but may be derived from central nervous system unidirectional maturational factors. Down’s syndrome may also exhibit asymmetry in teeth dimensions [12,13,25], as may other congenital conditions such as oligodontia [26].

### 1.3. Body Size as a Stressor

Numerous stressors, including the aforementioned injuries, hormones, and congenital conditions, can result in measurable asymmetrical biomarkers. Body size may also correlate with many potential stressors. The number of possible predators on an individual is often negatively correlated with their body size [27]. Larger animals tend to have larger home ranges [28,29], which may make them more sensitive to negative anthropogenic influences. Examples exhibiting this include gastropods [30,31], insects [32], and lizards [28,33,34]. Studies on certain reptiles suggest that positive anthropogenic influences may influence size, as urban lizards may be larger than their non-urban counterparts [35]. Other studies show that anthropogenic influences may have a neutral effect [36]. Another parameter to consider is food availability. Bigger individuals have bigger food items available [37,38] that require increased effort to obtain [39]. This may apply both inter- and intra-specifically, and directly influence jaw size, skull size, and head dimensions [40].

Asymmetry studies have not analyzed any reptiles with as diverse a body size as the family Varanidae [41,42,43], which includes the largest extant lizard, *Varanus komodoensis*, the Komodo dragon. Here, we investigate teeth asymmetry in a range of varanid species and sizes. This study is novel because it is the first to measure teeth asymmetry in reptiles, a trait that reflects multiple forms of stress in other vertebrates. It is consistent with the pattern of morphometric left bias (eye asymmetry, digit asymmetry, supra- and infra-labial asymmetry) and tail injury cited above [18,19,21,22,23,24]. We specifically test the directional asymmetry (DA) in varanid populations, isolating it from fluctuating asymmetry (FA). FA reflects developmental instability [44] and can be induced by stress [45,46]. However, DA is considered functional, with a genetic basis [1,47,48,49]. We hypothesized that the bigger the lizard species, the fewer potential predators and more potential food items it would have. Less stress would result from this, and consequently there would be less left-biased asymmetry. This would be similar to what is found in humans [50]. We therefore predicted to find more right-biased teeth asymmetry for bigger lizards.

## 2. Materials and Methods

### 2.1. Materials

Data were collected from dry skeletal specimens housed at the American Museum of Natural History, New York, NY (AMNH); Australia Museum, Sydney (AM); Field Museum of Natural History, Chicago, IL (FMNH); Florida Museum of Natural History, Gainesville, FL (FLMNH); Smithsonian Institution National Museum of Natural History, Washington, D.C. (USNM); and University of Michigan Museum of Zoology, Ann Arbor, MI (UMMZ). *Varanus* is a very large genus with numerous sub-genera and approximately 80 species over a large size range [41,51,52]. Specimens of *Varanus* were limited to those available within the collections, and, of these, specimens were chosen based on their number of measurable teeth and if they had representatives on both sides. Those teeth that did not have prominent cracks or chips that interrupted the margins were considered measurable. It was difficult to determine the degree to which wear influenced tooth shape, so only those with suspected light wear were included. Teeth that were not fully ankylosed, or not fused to the host bone, were not considered because they may have been immature or in the process of being shed. This resulted in 62 *Varanus* specimens from 13 species sampled, and, although this is a fraction of the overall diversity in the genus/family, it did represent the majority of the size range exhibited [41]. A total of 671 left/right pairs of teeth were ultimately measured (Table 1; Supplementary Materials).

### 2.2. Nomenclature

The nomenclature used here is standard reptile dental terminology [53,54]: mesial, towards the central premaxilla and mandibular symphysis; distal, away from the central premaxilla and mandibular symphysis; lingual, towards the tongue; labial, towards the lips; basal, towards the base of the tooth/where the tooth meets the host bone; apical, away from the host bone/towards the apex (if present).

The varanid specimens sampled here had a varied number of pleurodont tooth positions (Table 1), ranging from 4–5 along the premaxilla (often including a single, middle tooth position that was not measured), 9–13 along the maxilla, and 10–13 along the dentary. The number of tooth positions was determined by either the presence of teeth or empty indentations of the host bone surface.

### 2.3. Photography and Data Collection

Digital photographs of all teeth were taken from the labial perspective with an Olympus C-765 Ultra Zoom (Olympus Corporation, Toyko, Japan) or a Canon EOS T3 Rebel (Canon Inc., Tokyo, Japan) against a dark background. The camera was attached to a stand and positioned level, and each tooth was photographed separately. A scale bar was always included. For each tooth, we positioned the specimen so the camera lens was parallel to the host bone adjacent to the tooth. We simultaneously positioned the lens parallel to the apical–basal long axis, determined qualitatively as when the tooth looked its tallest to the photographer regardless of any labio–lingual curvature.

Varanid teeth have very few discrete homologous anatomical loci to collect linear measurements from, so we instead outlined the tooth to derive semi-landmarks. These are often used to define the contours of a tooth’s margin in both mammal molars [55,56,57,58] and reptile crowns [59,60,61,62,63]. Varanid teeth grow out beyond the parapet of the host bone on the lingual side, as is typical for pleurodont dentition. Measurements were taken from whatever portion of the tooth crown rose above this host bone margin. If the root was not visible from this perspective, the basal-most points of the marginal outline were defined as the junction between the host bone and visible tooth. If the tooth rose entirely above the margin of the host bone, the dentine of the root was visible and easily distinguished from the crown enamel; these teeth were traced along the margin of the crown and stopped at the root.

Photographs were entered in TpsDig 2.16, and the margin of the tooth was traced using the curve drawing tool [64]. For teeth with clear apices, this was traced from the apex to the base on both the mesial and distal sides. TpsDig then transformed each of the two traced margins into 30 equidistant coordinates, and we combined the apical-most coordinates. This resulted in 59 coordinates: 3 discrete landmarks (two at the base and one at the apex) and 56 semi-landmarks. For teeth without distinct apices, the entire margin of the tooth was traced from the basal-most point on the mesial side to the basal-most on the distal. This was also converted to 59 coordinates, with the end and the middle coordinates representing true landmarks and the rest semi-landmarks. This number of coordinates has been shown to accurately represent the totality of two-dimensional shape [65].

**Figure 1:**
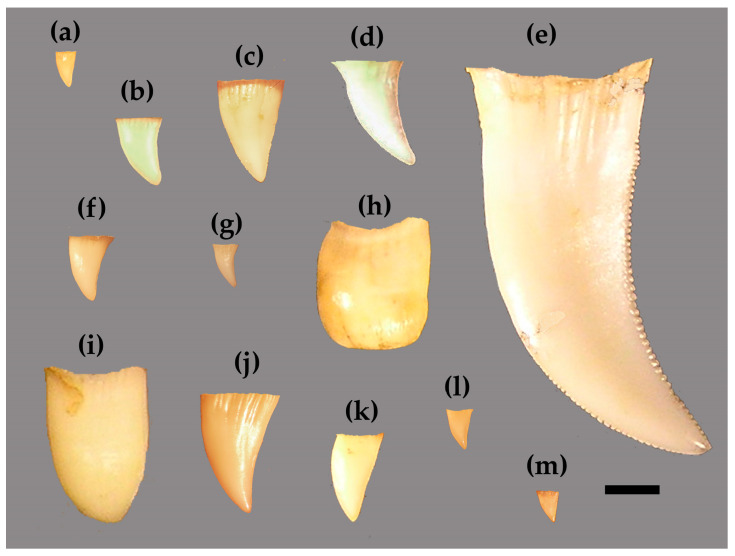
Examples of teeth from all *Varanus* species sampled in this study at the 6th position on the left maxilla. Scale bar = 2 mm. Species include (**a**) *V. acanthurus* (UMMZ 225497), (**b**) *V. dumerelli* (FLMNH 45210), (**c**) *V. gouldii* (UMMZ 210483), (**d**) *V. griseus* (FMNH 51705), (**e**) *V. komodoensis* (AMNH 37911), (**f**) *V. mertensi* (UMMZ 210485), (**g**) *V. mitchelli* (UMMZ 210489), (**h**) *V. niloticus* (AMNH 10500), (**i**) *V. olivaceous* (FLMNH 55169), (**j**) *V. panoptes* (UMMZ 210491), (**k**) *V. prasinus* (FLMNH 56949), (**l**) *V. scalaris* (UMMZ 210495), and (**m**) *V. tristis* (UMMZ 225519).

Conducting a semi-landmark analysis allowed us to ultimately derive a singular size metric: Centroid Size (CS) [66]. Varanid tooth crowns show a high degree of inter-specific shape variability, with variable degrees of height, thickness, lateral compression, curvature, and the presence/absence of serrations [59,67,68,69] (Figure 1). Euclidean linear measurements may be influenced by these factors. CS is a singular measure of size independent of shape, and eliminates the need to collect numerous measurements to account for shape variability. CS was calculated for each tooth using the program TpsRelw 1.53 [70], was is defined as the square root of the sum of squared distances of all the landmarks and semi-landmarks from their centroid.

### 2.4. Tooth Asymmetry Calculation and Statistical Analyses

Asymmetry was calculated using the formula: [(R − L)/(R + L)]. This was taken from van Valen [47]. Similar to Werner et al. [71], this calculation avoids an artificial equivalence in the scoring of asymmetry between different sized individuals, and the result is the proportion of the trait [6,7,71]. The CS calculation for the left (L) and right (R) tooth at each tooth position was plugged into the above equation, resulting in a single asymmetry calculation for every tooth position where left and right teeth were represented. A positive result indicated right-biased asymmetry, and a negative result was left-biased.

**Figure 2:**
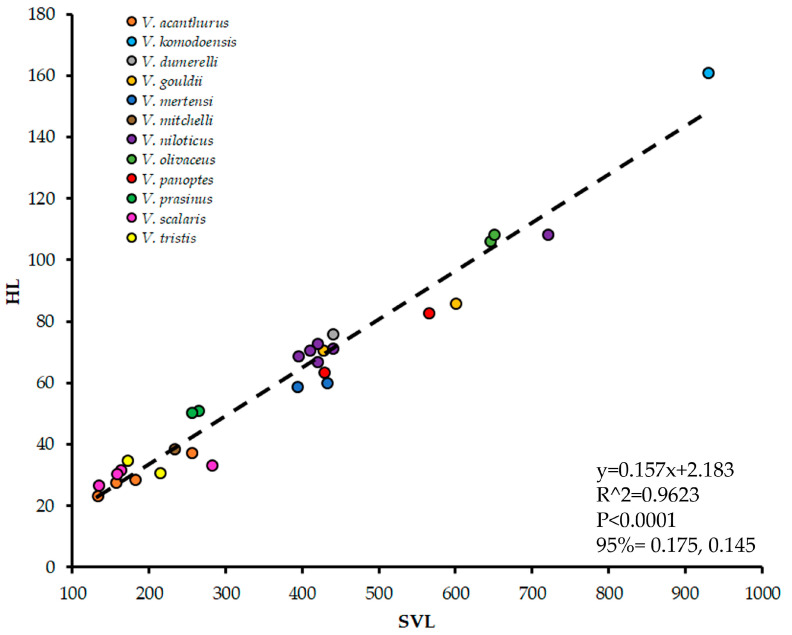
Body size allometry of snout-vent length (SVL) plotted against Head Length (HL) in millimeters for *Varanus* specimens where both forms of data were available. Regression information is depicted on the graph.

To offset the effect of FA, we used the rule that the average FA is zero at the population level [44]. We treat varanid dentition as populations in two ways: (1) all the tooth pairs in an individual lizard specimen were a single population, and (2) all the tooth pairs in all lizards of a single species were a single population. We then averaged these populations and normalized against body size to neutralize FA and show only DA. Unfortunately, commonly used metrics of body size, such as snout-vent length and mass [41], were recorded for only a portion of the specimens we collected data from prior to cataloging. Therefore, the length of the skull was used as a body size metric, as it was readily available for all specimens except two. This is referred to here as Head Length (HL). HL was taken from photographs of skulls using TpsDig 2.16 to determine the distance in millimeters between landmarks at the rostral-most point of the premaxilla and the caudal-most point on the supraoccipital along the mid-sagittal plane. We validate HL as a proxy for body size by regressing it against snout-vent length (provided in the label information in the 28 specimens where it is recorded, see Supplementary Materials) using an Ordinary Least Squared Regression Analysis (Figure 2) in PAST 5.1 software [72]. Indeed, HL was significantly correlated with snout-vent length (*p* < 0.05). As we could not collect HL for two specimens due to damage/disarticulation but had their snout-vent length, we then extrapolated it from the regression.

Several statistical analyses were conducted, all carried out in PAST. In order to test if the upper tooth row (premaxillary and maxillary teeth) exhibited significantly different (*p* < 0.05) tooth asymmetry from the lower (dentary teeth), we ran a two-tailed Student’s *t*-test comparing the two rows for all specimens. We then divided each tooth row into an anterior, middle, and posterior category, which resulted in 6 separate categories (premaxilla positions 1–4; maxilla positions 1–6; maxilla positions 7–13; dentary positions 1–4; dentary positions 5–8, and dentary positions 9–13). We then ran a One-Way Analysis of Variance (ANOVA) to determine if significant differences (*p* < 0.05) existed between these categories. Lastly, we tested the potential correlation between body size and our two “populations”. These two types of population were regressed against HL using an Ordinary Least Squared Regression Analysis. Analyzing average HL against average teeth asymmetry, using linear regression and Cook’s distance, suggested that *V. komodensis* was an extreme outlier (Cook’s Distances > 1 = 4.318). Thus, we removed the data for this species from the regression analysis to examine whether the model was sensitive to the effects of *V. komodensis*.

## 3. Results

### 3.1. Tooth Asymmetry Based on Position

Comparing the asymmetry of the upper and lower tooth rows resulted in a significant difference from the *t*-test (*df* = 669, *p*_Two-Tailed_ = 0.0416). The mean for the upper tooth row (0.0062 ± 0.0448) showed more right bias than the lower (−0.0005 ± 0.0395). When the tooth rows were subdivided, there was no significant difference between them according to the ANOVA (*F*_5, 665_ = 1.194, *p* = 0.3104). This indicated that there was no more left- or right-biased asymmetry on any one portion of the jaw than any other for the entire varanid sample.

### 3.2. Asymmetry and Body Size

**Figure 3:**
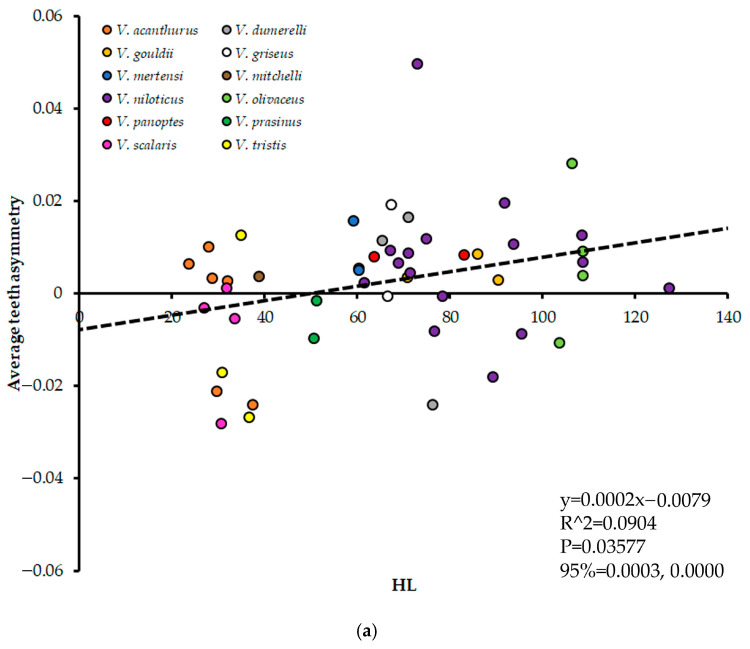

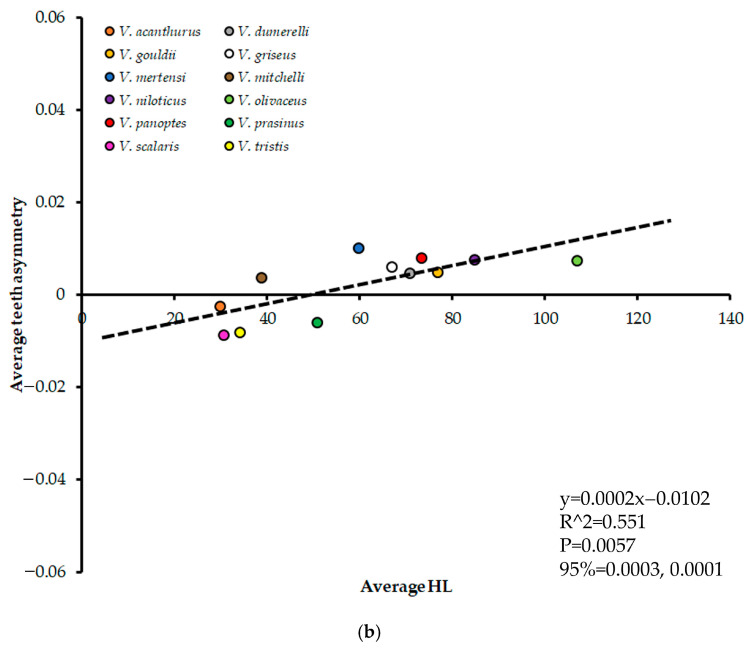
Body size in Head Length (HL) plotted against average teeth asymmetry for all *Varanus* species except *V. komodoensis*. (**a**) represents each lizard specimen as a single data point, and (**b**) represents values for all lizards of each species averaged together. Regression information is depicted on each graph.

When all species were considered, HL and asymmetry were not significantly correlated (*p* > 0.05), which was the case both when each individual lizard was regressed (*p* = 0.849) and when they were averaged by species and regressed (*p* = 0.218). The correlation became significant when excluding *V. komodoensis* though (*p* < 0.05), for both iterations of the data (Figure 3). Varanids with smaller HL exhibited left-biased asymmetry, and, as HL increased, teeth asymmetry became generally more right-biased. There was a high degree of scatter, with a single *V. niloticus* specimen acting as a noticeable outlier.

**Figure 4:**
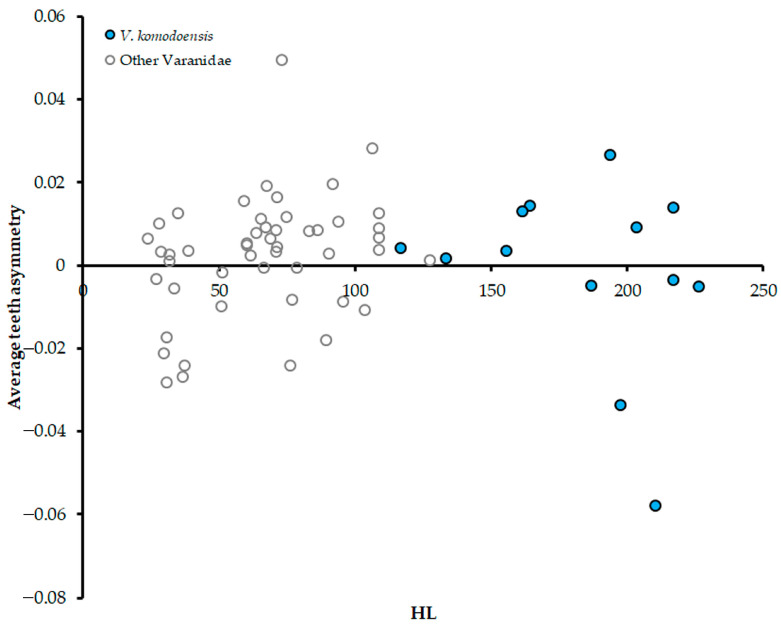
Body size in Head Length (HL) plotted against average teeth asymmetry all *V. komodoensis* specimens compared to remaining varanids. No significant regression was yielded for either *V. komodoensis* alone or all varanids together (*p* > 0.05).

When analyzing *V. komodoensis* separately, specimens with an HL under 180 mm appeared to trend along a similar trajectory to the other varanids in our sample (Figure 4). Once specimens went beyond that HL though, their asymmetry scattered noticeably. Above 180 mm, there are some right-biased *V. komodoensis* individuals mixed with strongly left-biased ones, some of which were more left-biased than any other varanids in our sample. To explore this further, we regressed solely *V. komodoensis* asymmetry against HL and a non-significant trend resulted (*p* = 0.364). We also divided the *V. komodoensis* sample into two groups, one with HL > 180 mm and one < 180 mm, and regressed each of their asymmetry against their HL. Once again, no significant trends were observed, but goodness of fit was relatively high for the smaller group (<180 mm group *R*^2^ = 0.511, *p* = 0.175; >180 mm group *R*^2^ = 0.006, *p* = 0.856).

## 4. Discussion

We found that the teeth asymmetry became significantly more right-biased as the varanid species increased in size, as long as the largest species was excluded. This was consistent with our hypothesis, and suggests that the stressors that induce asymmetry in the populations decrease as body size increases. We interpret teeth asymmetry as similar to other morphometric left-dominant asymmetries correlated with environmental stressors in reptiles: digit asymmetry and tail injury in lizards [1,4] and in *Sphenodon* [2], and eye asymmetry in snakes [5] and lizards [6,7,73]. This trend occurred inter-specifically here, but it is similar to intra-specific results in humans [50].

Smaller *V. komodoensis* individuals also showed an apparent increase in right-biased asymmetry as they increased in size like the rest of Varanidae, which was indicated by a relatively high goodness of fit. Once lizards reached a certain size though, the data scattered dramatically, with many specimens having more left-biased asymmetry (sometimes extremely so). As *V. komodoensis* is the biggest varanid species, members progress through a large range of sizes throughout ontogeny [74]. There is also ontogenetic ecological niche differentiation between young and mature individuals [67], from arboreal, insectivorous young to ground-dwelling, carnivorous adults. Similar life histories are not uncommon in reptiles, has been observed in crocodylians such as *Alligator mississippiensis* [75], and is hypothesized for theropods [76]. In *V. komodoensis* in particular, bigger individuals may have a larger home range. Their home ranges have been recorded to contract in proximity to human settlements, indicating that *V. komodoensis* may suffer from anthropogenic influences in a different fashion than their congenerics [77]. In addition, studies have shown both negative and positive influences on their populations by ecotourism [78]. The shift in asymmetry in larger *V. komodoensis* individuals away from the trajectory of the rest of the clade could be indicative of implicit stressors associated with these niche and home range shifts. Another possibility is sexual dimorphism, as male *V. komodoensis* are substantially larger than females [67]. As testosterone is a stress hormone in lizards [11] and fighting in male *V. komodoensis* is common [67], larger males may reflect more left-biased asymmetry. If the largest specimens in our data set were male, this may be the driver (sex data were unfortunately not available for almost all of our specimens). Any of these explanations potentially justify the exclusion of *V. komodoensis* from the remainder of Varanidae concerning the relationship between asymmetry and body size. Although any directional trends of asymmetry in *V. komodoensis* were found to be non-significant when the sample was divided up, averaging teeth asymmetry to yield DA diminished our sample size, making reliable significance difficult to obtain. This study may therefore be considered as a pioneering pilot test, serving as “food for thought” when considering future research with a larger sample size.

Although the region of the tooth row did not vary in degree of asymmetry, there was a significant difference between the upper and lower jaws. The fact that the upper jaw was more right-biased may indicate that it is less susceptible to the asymmetric effects of stress than the lower. Mandibular asymmetry influencing tooth development has been seen in modern crocodylians [79], so it is possible that this may also influence Varanidae. These correlative effects may both result from similar stressors, and future studies should consider jaw dimensions as a potential biomarker in these taxa.

It is possible that, when FA is negated, the DA of teeth is a biomarker for brain laterality. Many major biomarkers of asymmetry commonly correlate with brain laterality [6], and may often be a consequence of prenatal stress [80,81]. Effects of brain laterality include digit ratio asymmetry [3,4,7,71,82], digit asymmetry [7,73], and eye asymmetry [5,6,7,73]. Handedness and footedness is derived from brain laterality [3,7], is reflected in gyrification asymmetries [83], and is linked to testosterone level and risk-taking strategy [3,7]. The dominant hand usually has greater size and grip [83], possibly due to bigger muscles and frequency of use. Brain laterality can also be linked to footedness-indicated detour-tests [3,7,71]. In lizards within a population, digit ratios and high-risk behaviors together reflect brain laterality [3,7]. This is manifested in higher risk-taking strategies for the left-biased and risk averse strategies for the right-biased at the population level, as demonstrated for humans, birds, and lizards [84]. Teeth asymmetry may be less intuitively associated with laterality than, for example, handedness, but further study should focus on this as a potential direct mechanism.

Another aspect to be further investigated is what specifically causes the size asymmetry seen in varanid teeth. The effect of wear on teeth asymmetry is explained by asymmetric feeding behavior, wearing more extensively the dominant side in both humans [22] and extinct durophagous reptiles [23]. However, we suspect that teeth asymmetry is most likely not derived from asymmetric behavior. Harris and Bodford [20] claimed that prior studies attributed this sidedness to compensations for hemispheric laterality (but cited none). However, emergence and eruption characters are believed to be linked with prenatal and early postnatal growth and development [24]. In addition, reptiles are polyphyodont and constantly shed their teeth [85]. This may reduce the effect wear has on morphology, as the teeth may be shed before wear makes a noticeable impact.

The take-home message from these results is that the inter-specific teeth asymmetry in many varanid lizards has a significant positive trend with body size. Besides the FA component, there is also a functional DA component that should not be ignored. Tooth DA can be used as a biomarker at the population level to measure environmental stress, similar to other known stress-biomarkers in reptiles.

## Figures and Tables

**Table 1:** All species of *Varanus* considered in this study, along with the number of specimens sampled for each. “Tooth pairs” indicates the number of left and right teeth used to determine asymmetry in each species. “Tooth positions” indicates the number of possible positions that teeth may be found at per each host bone (premaxilla = P, maxilla = M, dentary = D), excluding the singular middle premaxillary position if present.

*Varanus* Species	Specimens	Tooth Pairs	Tooth Positions
*V. acanthurus*	6	60	P = 3, M = 9, D = 10
*V. dumerelli*	3	43	P = 3, M = 9, D = 10
*V. gouldii*	4	55	P = 4, M = 11, D = 12
*V. griseus*	2	12	P = 4, M = 10, D = 11
*V. komodoensis*	13	101	P = 4, M = 13, D = 13
*V. mertensi*	2	21	P = 4, M = 12, D = 12
*V. mitchelli*	1	10	P = 3, M = 10, D = 11
*V. niloticus*	16	209	P = 4, M = 11, D = 12
*V. olivaceus*	4	50	P = 4, M = 10, D = 12
*V. panoptes*	2	16	P = 4, M = 12, D = 12
*V. prasinus*	2	24	P = 4, M = 10, D = 11
*V. scalaris*	4	40	P = 4, M = 10, D = 10
*V. tristis*	3	30	P = 4, M = 10, D = 10

## Data Availability

Spreadsheets of varanid specimens, centroid sizes, and body size data and extrapolation are available in Supplementary Material.

## Supplementary Materials

The following supporting information can be downloaded at: https://www.mdpi.com/article/10.3390/jdb13010009/s1. Spreadsheet of (1) Head Length and snout-vent length (when available) for all varanid specimens, (2) Centroid Size and dental asymmetry for all left-right pairs of teeth, and (3) the average dental asymmetry for all teeth in an individual specimen for all specimens. Tooth positions were numbered in ascending order starting at the mesial-most position of each host bone, and the host bone was symbolized by the first letter (premaxilla = P, maxilla = M, dentary = D).
